# Spiny mouse (*Acomys*): an emerging research organism for regenerative medicine with applications beyond the skin

**DOI:** 10.1038/s41536-020-00111-1

**Published:** 2021-01-04

**Authors:** Janak Gaire, Justin A. Varholick, Sabhya Rana, Michael D. Sunshine, Sylvain Doré, W. Brad Barbazuk, David D. Fuller, Malcolm Maden, Chelsey S. Simmons

**Affiliations:** 1 Department of Mechanical and Aerospace Engineering, University of Florida, Gainesville, 32611 USA; 2 Department of Molecular Genetics and Microbiology, University of Florida, Gainesville, 32611 USA; 3 Department of Physical Therapy, University of Florida, Gainesville, 32611 USA; 4 Department of Anesthesiology, University of Florida, Gainesville, 32611 USA; 5 Department of Biology and UF Genetics Institute, University of Florida, Gainesville, 32611 USA; 6 McKnight Brain Institute and Center for Breathing Research and Therapeutics, University of Florida, Gainesville, 32611 USA; 7 J. Crayton Pruitt Family Department of Biomedical Engineering, University of Florida, Gainesville, 32611 USA

**Keywords:** Physiology, Preclinical research, Regeneration and repair in the nervous system, Experimental models of disease

## Abstract

The spiny mouse *(Acomys* species*)* has emerged as an exciting research organism due to its remarkable ability to undergo scarless regeneration of skin wounds and ear punches. Excitingly, *Acomys* species demonstrate scar-free healing in a wide-range of tissues beyond the skin. In this perspective article, we discuss published findings from a variety of tissues to highlight how this emerging research organism could shed light on numerous clinically relevant human diseases. We also discuss the challenges of working with this emerging research organism and suggest strategies for future *Acomys*-inspired research.

## Introduction

Damaged tissues or organs, especially in adult mammals, do not regenerate and are instead replaced by a dense scar through processes collectively known as fibrosis. Chronic fibrosis can affect tissues throughout the body, ultimately leading to organ failure and death. Annually, millions of people worldwide lose their lives to fibrosis and, in the United States, fibrosis-related deaths account for approximately 45% of all deaths^1^. To address this, the field of regenerative medicine has emerged with a hope to replace and restore the structural and functional integrity of the damaged tissue for individuals suffering from debilitating conditions.

**Fig. 1:**
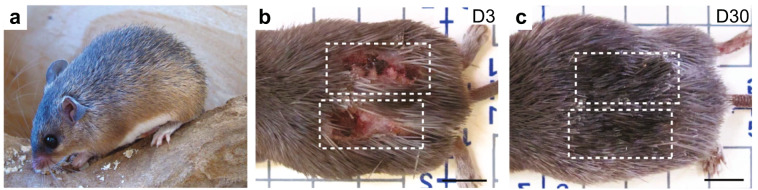
Skin wounds heal and hairs regrow completely in *Acomys*. *Acomys kempi* (**a**) rapidly form scabs after full-thickness skin injury at day 3 (D3, **b**), and the same wounds are concealed with new skin and spiny hairs at day 30 (D30, **c**) Scale bars, 1 cm. Image adapted from Seifert et al.^3^ with permission.

Invertebrates, fish, and amphibians with regenerative capabilities have traditionally served as research organisms for regenerative biology. While regeneration has also been documented in higher-order organisms, it is mostly limited to fetal/neonatal healing and select tissues, e.g., liver. While insights gleaned from these regenerative systems have contributed to our understanding of developmental and stem cell biology, the translational impact remains limited. Recently, spiny mice (genus: *Acomys*^2^) have emerged as an exciting new organism for research in regenerative medicine. Several species of *Acomys* are typically found in dry-arid conditions of the Middle East, South Asia, and parts of Africa, and local populations traded stories of *Acomys* species’ autotomic tail “degloving” for generations. The legend of *Acomys* was perpetuated globally as certain species became attractive exotic pets, and the regenerative capacity of *Acomys* was first experimentally documented by Seifert et al. (Fig. 1)^3^. Subsequent studies have confirmed the remarkable ability of at least three *Acomys* species to fully regenerate and regrow complex tissues such as full-thickness skin, ear tissue, and skeletal muscle^3–6^.

Observational reports of field-caught *Acomys* appear at the beginning of the 20th century, and biomedical research studies of *Acomys* increase in number after the 1950s. Early studies with *Acomys* largely focused on physiological adaptations related to desert-like conditions and their sensitivity to developing diabetes when fed a high-fat or high-sugar diet^7,8^. In addition to their propensity toward acquired diabetes, *Acomys* exhibit other human-like qualities that are rare in rodents; namely, they complete nephrogenesis before birth^9^, produce steroid hormones in their adrenal glands^10^, and are known to menstruate^11^. *Acomys* have also been commonly used in developmental research because of their precocial development and long gestation period (i.e., 40 days) compared to other common rodents^12^. Given these unusual features of *Acomys* biophysiology that often parallel humans, the remarkable ability of *Acomys* to undergo scarless regeneration makes them all the more attractive as a research organism for regenerative medicine. In this perspective review article, we discuss recent findings of *Acomys* regeneration and describe opportunities and challenges of future research in the hopes of expanding related research and, ultimately, helping patients recover from a wide range of acute and chronic conditions.

## Insights into regeneration from *Acomys* organ systems

Early insights into *Acomys* regeneration have come from dermal wound healing studies that include full-thickness biopsy punches of the skin and ear and thermal burns of the skin^4,5,13–16^. Dermal fibrosis in humans can occur as a result of an accidental injury, life-saving surgery, or systemic fibrotic disease like scleroderma, severely affecting the functionality of the damaged area and the overall well-being of the patient. While progress has been made in mitigating scar tissue, complete restoration (i.e., regeneration) of skin with intact hair and glands remains a clinical challenge. After injuries mimicking human trauma, *Acomys* skin undergoes rapid re-epithelization and regrows with hair follicles, dermis, glands, and muscle through the continued proliferation of cells^3–5,13,14,16,17^. In addition to full-thickness skin regeneration, ear holes in *Acomys* made via full-thickness biopsy punch fully close with newly formed blood vessels, cartilage, muscle, and nerve fibers occupying the regenerated region^4^. Though closure of ear hole punches has been well documented in rabbits and a select strains of immunodeficient mice^14,18–20^, most mouse species, including one sympatric with *Acomys*, do not regenerate after ear punches^14^. 2 mm ear hole punches has been reported to close in Murphy Roths Large (MRL/MpJ) mice, a strain known as “super healers”, but they do not fully close large (>4 mm) ear holes^14^ nor do they heal full-thickness excisional wounds^21,22^. Similarly, FOXN1-deficient (nude) mice heal incisions without scarring^18^, but regenerative properties, e.g., after large wounds, have not been characterized to date. Regeneration of large dermal wounds via extensive proliferation of multipotent cells remains unique to *Acomys* among adult mammals, underscoring the utility of *Acomys* as a research organism to investigate mechanisms underlying dermal wound healing.

While the mechanisms behind *Acomys* skin regeneration remain elusive, initial comparative studies of *Acomys* and *Mus*, a standard laboratory mouse, suggest that immune cells play a central role in the orchestration of scar-less regeneration of *Acomys* skin and ear wounds. Following an injury, pro-inflammatory factors are downregulated and pro-reparative factors are upregulated in the *Acomys* wound bed compared to *Mus*^13,15,17,23^. Though inflammatory macrophages (M1-type) are minimal or absent in healing *Acomys* wounds, experimental depletion of all macrophages delays ear hole closure in *Acomys*^15^, reminiscent of failed regeneration in axolotl^24^ and mouse digit-tips^25^. Such experiments suggest that impaired regeneration may be related to the depletion of pro-reparative macrophages (M2-type) that are otherwise abundant in *Acomys* wounds^6,15^. *Acomys* also have lower neutrophils in comparison to *Mus*^5,26^, suggesting differences in other immune cell populations and function. Future *Acomys* studies including baseline characterization of tissue-specific immune cells and their relative contributions to various injury models could provide clinically-relevant insights that support intervention strategies to treat and manage fibrotic diseases.

Demonstration of scar-free regeneration of *Acomys* skin and characterization of immune cell responses have inspired researchers to explore a broad range of questions in regenerative medicine, including musculoskeletal, nervous system, cardiovascular, and renal regeneration.

### Musculoskeletal system

Musculoskeletal conditions such as volumetric muscle loss injury, sarcopenia, and osteoarthritis are the single highest contributor to global disability worldwide, with nearly a quarter of the world living with a painful musculoskeletal condition^27^. Musculoskeletal injuries often happen in conjunction with dermal wounds after trauma and can significantly impair a patient’s mobility, leading to an increased risk of chronic health conditions. While preclinical studies from small laboratory animals, such as rodents, and large farm animals, such as goats, pigs, sheep, and cows, have improved our fundamental understanding of disease pathophysiology, challenges in functional restoration of skeletal muscle have limited progress towards improving patient health and well-being.

**Fig. 2:**
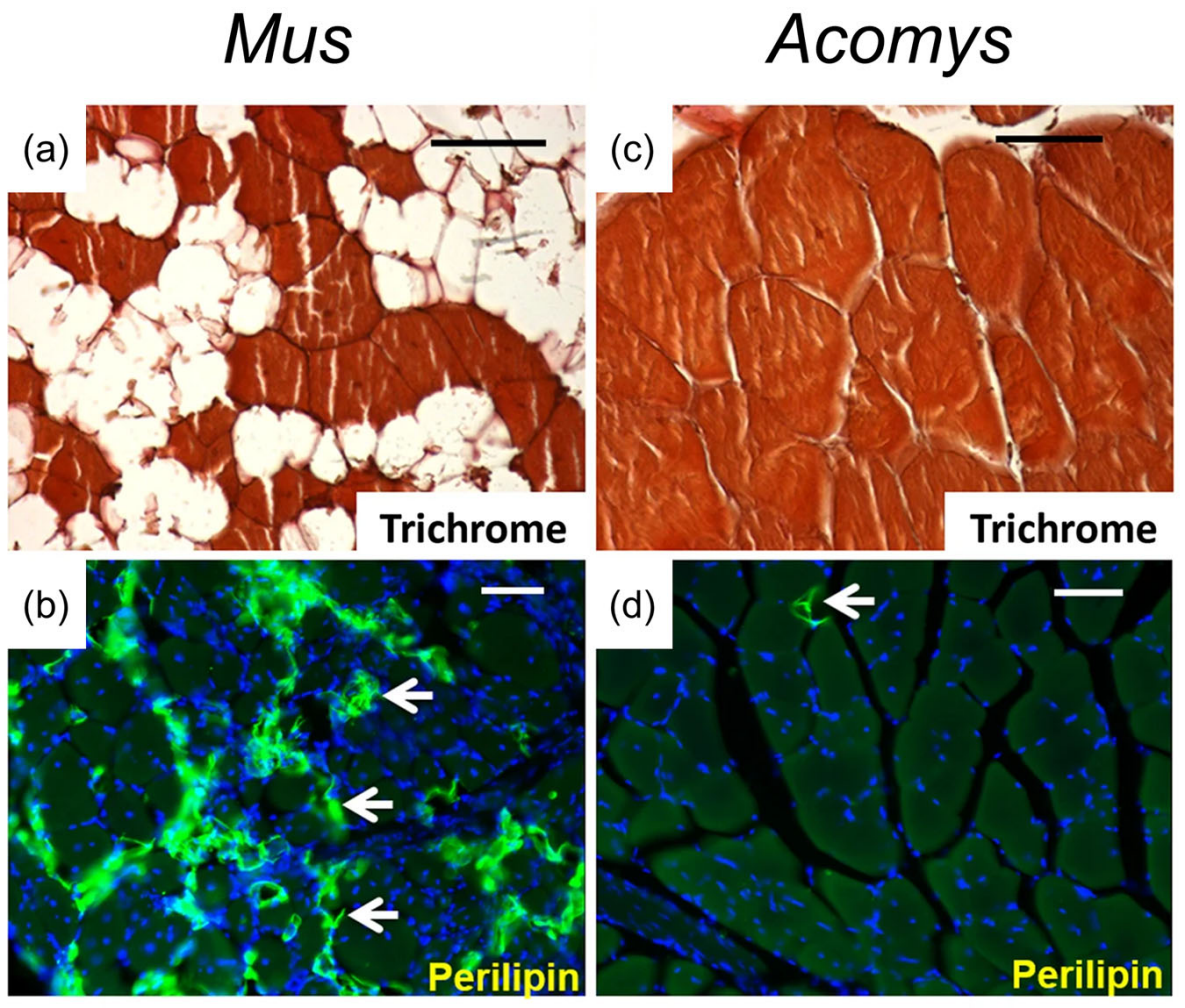
Muscle regeneration in *Acomys*. Representative images of *Mus* (**a**, **b**) and *Acomys* (**c**, **d**) muscle after repeated injections of myotoxin. *Acomys* muscle regenerates perfectly and looks similar to uninjured tissue whereas in *Mus*, myofibrils are replaced with adipose tissue. Accumulation of adipose tissue in *Mus* is reflected in white regions of Trichrome stain (**a**, scale bar = 50 µm) and immunolabeled perilipin, a marker for adipocytes as indicated by white arrows (**b**, scale bar = 100 µm). Green = perilipin, blue = nuclei. Image adapted from Maden et al.^6^ under Creative Commons Attribution 4.0 International License.

To date, the remarkable regeneration of skeletal muscle has been recorded in *Acomys*. For example, the tibialis anterior regenerates in *Acomys* at a faster rate than in *Mus* after a single injection of myotoxin, a peptide found in snake venoms leading to muscle necrosis^17^. Further, after repeated injections of myotoxin, *Mus* failed to regenerate and replaced myofibers with adipose cells instead, whereas *Acomys* repeatedly regenerated in a consistent fashion, demonstrating its superior regenerative properties (Fig. 2)^6^. In addition, unlike in *Mus*, the panniculus carnosus layer of skeletal muscle beneath the hypodermis in rodents regenerates in *Acomys* 5 weeks following injury. Some studies have also reported that the skeletal muscle of ears regenerates after 4 mm ear hole punches^4^. Additional transcriptomic studies of skin wounds revealed that 32 of the top 50 differentially expressed genes in *Acomys* were directly related to muscle development and function, and embryonic myosin was induced 450-fold^23^, suggesting the activation of myogenic pathways similar to fetal development. These observations support the notion that volumetric muscle loss after injury in adult mammals can be recovered, warranting additional *Acomys* studies to probe into underlying mechanisms. The fact that *Acomys* muscle and ear cartilage possesses superior regenerative ability as compared to *Mus* also opens the door for studying additional musculoskeletal conditions associated with joints and ligaments.

### Central nervous system

Functional recovery after damage to the mammalian central nervous system (CNS) notoriously presents a profound challenge to clinicians due to the remarkably poor capacity of neurons to regenerate, and an estimated 80 million individuals globally are living with disability from traumatic brain and spinal cord injuries^28^. To tackle this problem, physiologists and neuroscientists have often taken a comparative approach by examining how regeneration-competent invertebrates and non-mammalian vertebrates achieve superior neural regeneration. Unlike humans, spinal cord injury in the axolotl salamander (*Ambystoma mexicanum*) can be fully repaired with functional reconnection of the rostral and caudal parts of the injured spinal cord^29^, possibly because glial cells support a regenerative state^30^. The adult zebrafish (*Danio rerio*) also shows the ability to regrow the brain, spinal cord, and retinal tissue from resident glial cells following lesions^31,32^. However, to date, there has been extremely limited success with preclinical testing of mammalian regeneration strategies gleaned from these non-mammalian models.

**Fig. 3:**
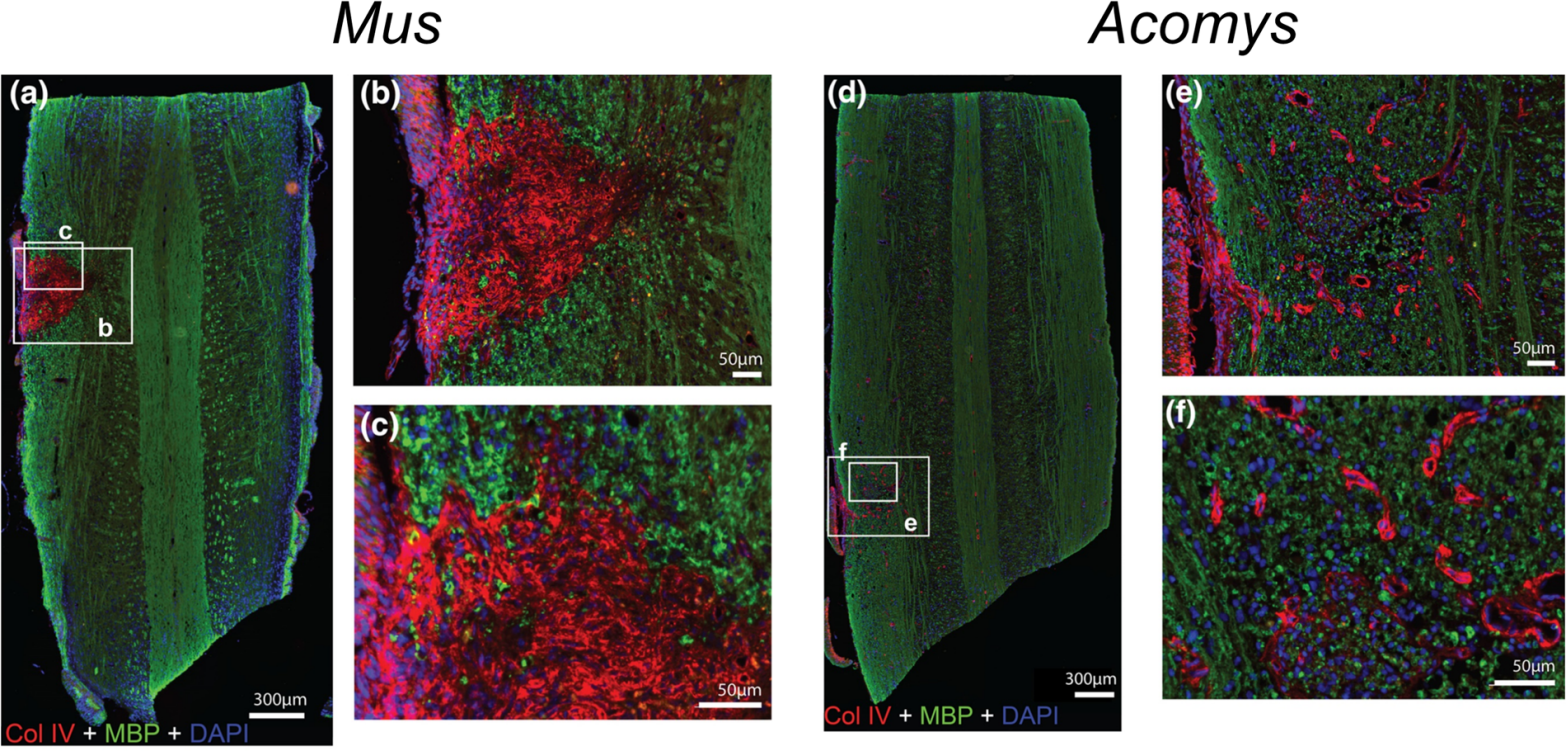
Minimal fibrosis is observed in *Acomys* following spinal cord injury. Representative images of *Mus* (**a**–**c**) and *Acomys* (**d**–**f**) cervical spine, 4 weeks post-spinal cord injury. High magnification images of the lesion epicenter show excess collagen IV deposition in *Mus* (**b**, **c**) in contrast to cell growth in *Acomys* (**e**, **f**). Scale bars in **a** and **d** = 300 µm and **b**, **c**, **e**, and **f** = 50 µm. ColIV = Collagen IV, labeled red; MBP = myelin basic protein, a marker for oligodendrocytes, labeled green; DAPI = nuclei, blue. Image reprinted from Streeter et al.^33^ with permission.

*Acomys* has emerged as an up-and-coming experimental mammalian model for CNS regeneration. Following a spinal cord injury, *Acomys* showed a very unique molecular and immunohistochemical response in the injured spinal cord and more rapid recovery of bladder function as compared to the commonly studied laboratory mouse (*Mus*, C57BL/6 strain). The unique responses included significantly increased expression of neurogenesis-related genes, as well as reduced histological evidence of fibrosis (Fig. 3)^33^, indicating that *Acomys* is a useful research organism to study adaptive responses to spinal injury. Future studies of mechanisms underlying reduced gliosis and its functional consequences will provide essential clues on CNS regeneration.

### Renal system

Renal fibrosis, a hallmark of many renal disorders, including chronic kidney disease, is a debilitating complication affecting millions of people worldwide^34^. Currently, patients with advanced kidney disease must undergo routine dialysis or receive organ transplantation, both of which are inaccessible for many patients. Similar to other fibrotic diseases, tremendous efforts have been employed to understand and intervene in renal fibrosis, but the intricacies of kidney structure and pathophysiology complicate efforts to recapitulate disease conditions and develop therapeutic interventions.

Recently, Okamura et al. demonstrated that the regenerative ability of *Acomys* extends to the kidneys^35^. Specifically, restoration of both the structure and function of the kidney was reported following acute and chronic models of aggressive kidney injury. Moreover, as with skin and muscle regeneration, minimal fibrosis and reduced inflammation-associated markers were observed in obstructed kidneys, including at chronic time points. Interestingly, unlike in other rodents, nephrogenesis occurs before birth in *Acomys*^9^, making it a valuable organism to study kidney development and pathophysiology in utero for maternal-fetal considerations and congenital defects. Understanding the mechanisms underlying restoration of structure and function to *Acomys* kidneys could inspire novel therapeutic approaches for patients with severe renal diseases, including chronic kidney disease associated with the rising incidence of type 2 diabetes and high blood pressure^34^.

### Cardiovascular system

Cardiovascular disease remains the leading cause of death in the United States and is increasing globally^36^. In many cardiovascular diseases, including heart attacks and stroke, oxygen transport to target tissues becomes limited, which permanently damages the tissue. While damaged cardiac tissues in zebrafish fully recover via the proliferation of cardiomyocytes^37^, higher-order animals including most adult mammals cannot recover. However, preliminary reports suggest *Acomys* can regenerate the heart after oxygen-deprivation-induced cardiomyopathy. At baseline, *Acomys* and *Mus* have similar heart weight to body weight ratios and ejection fractions^38^. After coronary artery ligation, however, *Acomys* ejection fraction recovered (unlike in *Mus*), and more actively proliferating cardiomyocytes were present in *Acomys* than in *Mus*^39,40^.

In addition to understanding pathways that control cardiomyocyte proliferation, *Acomys* may provide clues to avoiding hypoxic damage altogether. Non-traditional research organisms such as the naked mole-rat have previously proven useful to study mechanisms of low oxygen (hypoxia) in mammals^41^. In addition, injecting stem cells can improve cardiac function in *Mus* post-injury by activating an acute immune response as characterized by the induction of CCR2+ and CX3CR1+ macrophages^42^. If the innate immune system of *Acomys* can effectively regulate inflammation post-injury, understanding these mechanisms of *Acomys* regeneration may offer insight into the prevention and reversal of heart disease.

## Maintaining *Acomys* as a research organism

While recent findings in *Acomys* are intriguing, additional work is necessary to unravel the mechanisms underlying adult mammalian regeneration for eventual translation into clinics. We foresee limited availability of *Acomys* (species and numbers) for the broader research community as a major hurdle for widespread research applications. To date, *Acomys* are not maintained by vendors such as Charles River or the Jackson Laboratory; rather, small colonies are maintained by a handful of researchers for their individual projects. Procurement of *Acomys* is currently done by agreements between individual researchers and their institutions, limiting the widespread use of *Acomys* among researchers with diverse research interests. With growing enthusiasm for studying *Acomys*, a strategic plan to establish and disseminate various *Acomys* species (e.g., *subspinosus, spinossisumus, russatus, wilsoni*, and *cahirinus*)^2^ in large numbers, including the three species (*A. percivali*, *A. kempi*, and *A. cahirinus*) that have been proven to regenerate, would significantly benefit the biomedical research community.

Maintaining *Acomys* colonies require adjustments from traditional processes for laboratory rodents. Compared to regular laboratory mice, *Acomys* take longer to reach sexual maturity (2–3 months of age), have a more extended gestational period (approximately 40 days), and produce small litters of 1 to 3 pups, all of which make it difficult to maintain and build a colony with a large number of animals. Housing conditions that increase breeding also differ from standard mouse protocols, including extra space, a protein-rich diet, additional environmental enrichment, and a higher temperature (~26 °C). In addition to environmental considerations, *Acomys* have weak skin and tail autotomy, so researchers must adopt special handling and restraining techniques for routine husbandry practices, injections, and behavioral assessments^43^. Example modifications include (a) the use of hand towels to restrain for identification of sex, subcutaneous injections, and ear tattooing, (b) the use of plastic restraint bags while collecting bodily fluids such as urine and measuring vitals such as body temperature, and (c) the use of plastic cups for transferring. Additional considerations for *Acomys* care and husbandry are nicely discussed in recent reviews^43–45^.

## Outlook for *Acomys* as a research organism

Although the use of *Acomys* species for observational studies dates back to the early 20th century, modern demonstrations of its unique ability to regenerate a range of adult tissues have reinvigorated enthusiasm for *Acomys* as a research organism for a wide range of clinically relevant diseases. One of the features potentially underpinning *Acomys* regeneration is an altered immune response. Previous studies across a variety of injury models suggest that a blunted inflammatory response in the *Acomys* wound bed facilitates regeneration^5,15,23,33,46^; however, the exact role, timing, and interplay of immune cells, including various macrophage phenotypes, are not well understood. Cultures of primary cells in combination with transgenic approaches to knock down or silence genes of interest are likely to yield insights more quickly than whole-organism modification, given the complexities of *Acomys* reproductive biology. In vitro experiments also afford scalability for higher throughput assays to investigate mechanistic and functional implications of a particular signaling pathway. Since stromal cells from *Acomys* also exhibit unique anti-fibrotic behavior^47^, testbeds to isolate independent functions of specific cell types could be advantageous for translational applications. Interactions among multiple cell types, such as the interplay between parenchymal, immune, and stromal cells, could be parsed out using innovative co-culture testbeds. Taking a step further, in vivo systems such as engineered chimeras and transgenic mouse lines could also be useful to unravel underlying cellular and molecular mechanisms of mammalian regeneration.

In addition to the challenges associated with breeding and colony maintenance discussed in section 3, there are methodological challenges to take into consideration before commencing *Acomys* studies. As its common name suggests, spiny mice were initially grouped under the *Murinae* subfamily with regular mice and rats; however, it is becoming increasingly apparent that *Acomys* “mice” are not the same as traditional laboratory mice and rats. In practice, this distinction is impactful because molecular reagents and resources developed for common rodent research organisms often do not work well on *Acomys* species. Empirical evidence that surface markers used for immunophenotyping mice and/or human cells fail to react with *Acomys* cells^46,48,49^ is supported by molecular and evolutionary findings from the 1990s, providing evidence of *Acomys*’ proximity to the Gerbillinae subfamily that comprises Mongolian gerbils^50,51^ and other specialized East African rodents^52^. Therefore, to draw helpful conclusions from *Acomys*-related studies, additional control experiments, along with careful validation of existing molecular tools, are warranted. Furthermore, efforts should be made towards developing *Acomys*-specific molecular reagents.

Furthermore, many molecular assays (e.g., PCR) are predicated on the availability of *Acomys* genome information. Several recent studies have resulted in RNA-Seq transcript sequence collections from *Acomys* to explore gene content^33^ and differential gene expression within *Acomys* and in comparison to non-regenerative species^14,53^. While providing a rapid and inexpensive source of expressed sequence data, short-read transcriptome assemblies are often fragmented, and transcript representation is biased by expression level and by the tissue type sampled. Using de novo assembled transcriptomes as a reference for RNA-Seq expression studies is often complicated by miss-assemblies that can underrepresent the true extent of expression for a given locus. In addition, *Acomys*-*Mus* orthologue assignments can be confounded by miss-assemblies and the inability to consult conserved synteny. To address these inadequacies and provide a foundation for future genomics analyses by the *Acomys* regeneration community, a whole-genome reference sequence is desirable. Toward this end, researchers at the University of Florida have recently sequenced and assembled an *Acomys* reference genome using PacBio long-read sequences with support from the National Institutes of Health. Approximately 60X redundant coverage of the *Acomys* genome (expected size ~2.3 Gb^54^) in PacBio reads were obtained from high molecular weight DNA extracted from the liver dissected from a single male. This reference genome is currently being annotated prior to public release in Winter 2020. This will enable the development of *Acomys*-specific molecular reagents and tools, thereby accelerating future *Acomys* studies.

In conclusion, the regenerative capability of *Acomys* species across a wide range of tissues is intriguing, and they undoubtedly hold considerable promise from a translational standpoint. However, additional work is necessary to identify mechanisms underlying scar-less regeneration. Recent technological advancements in molecular and cell biology techniques, including CRISPR and single-cell RNA-seq, promise to advance our understanding of the regenerative power of *Acomys*. Supported by a growing community of researchers and innovative methods, *Acomys* is an increasingly powerful tool for the regenerative medicine community to leverage in its goal of restoring structure and function to damaged tissues and improving quality of life for people with devastating medical conditions.
